# Effects of SiC and Resorcinol–Formaldehyde (RF) Carbon Coatings on Silicon-Flake-Based Anode of Lithium Ion Battery

**DOI:** 10.3390/nano11020302

**Published:** 2021-01-25

**Authors:** Yonhua Tzeng, Jia-Lin He, Cheng-Ying Jhan, Yi-Hsuan Wu

**Affiliations:** Department of Electrical Engineering, Institute of Microelectronics, National Cheng Kung University, One University Road, Tainan City 70101, Taiwan; jackson741t12@gmail.com (J.-L.H.); m10506126@gmail.com (C.-Y.J.); d980372@gmail.com (Y.-H.W.)

**Keywords:** silicon, SiC, LIB, anode, graphitic carbon, Resorcinol–Formaldehyde

## Abstract

Silicon flakes of about 100 × 1000 × 1000 nm in sizes recycled from wastes of silicon wafer manufacturing processes were coated with combined silicon carbide (SiC) and graphitic (Resorcinol–Formaldehyde (RF)) carbon coatings to serve as active materials of the anode of lithium ion battery (LIB). Thermal carbonization of silicon at 1000 °C for 5 h forms 5-nm SiC encapsulating silicon flakes. SiC provides physical strength to help silicon flakes maintain physical integrity and isolating silicon from irreversible reactions with the electrolyte. Lithium diffuses through SiC before alloying with silicon. The SiC buffer layer results in uniform alloying reactions between lithium and silicon on the surface around a silicon flake. RF carbon coatings provide enhanced electrical conductivity of SiC encapsulated silicon flakes. We characterized the coatings and anode by SEM, TEM, FTIR, XRD, cyclic voltammetry (CV), electrochemical impedance spectra (EIS), and electrical resistance measurements. Coin half-cells with combined SiC and RF carbon coatings exhibit an initial Coulombic efficiency (ICE) of 76% and retains a specific capacity of 955 mAh/g at 100th cycle and 850 mAh/g at 150th cycle of repetitive discharge and charge operation. Pre-lithiation of the anode increases the ICE to 97%. The SiC buffer layer reduces local stresses caused by non-uniform volume changes and improves the capacity retention and the cycling life.

## 1. Introduction

### 1.1. High-Capacity Long-Cycling-Life Anode for Lithium Ion Battery

Lithium ion battery (LIB) is the most popular and ubiquitous rechargeable energy storage device in the modern society. It has contributed a great deal to human civilization by enabling mobile 3C devices and enriching human life. However, new and challenging demands continue to emerge. The anticipated electric vehicles and large-scale renewable energy storage are two examples. Electric vehicles with a long drive range require LIBs with high energy density and capacity. A renewable energy system, which usually generates electricity at a weather dependent rate, needs a large-scale energy storage system. To meet the demands, energy density, power density per weight and per volume of LIBs, and the discharge/charge cycling life require much improvement. However, the higher the energy density is, the more dangerous a large-scale battery system will be. Battery safety is at the highest priority especially for futuristic LIBs of very high energy and power densities [1,2,3,4,5,6,7,8,9,10,11,12,13,14,15,16,17,18,19]. A desirable LIB consists of optimized combination of anode, cathode, separator, electrolyte, and packaging. For critical applications, power management circuits are integrated with battery systems. Graphite is the main active material for modern LIBs because graphite is abundant, inexpensive, and electrochemically stable in a LIB system. Graphite-based anode is theoretically capable of storing less than 400 mAh/g of charges. It exhibits a long cycling life and high Coulombic efficiency (CE) including that in the very first cycle known as initial CE (ICE). Higher Coulombic efficiency means more inserted lithium is recycled. This is important because in a packaged LIB, there is a fixed amount of lithium. When lithium is consumed to a low level, LIB fails prematurely. However, the low specific capacity of graphite-based anode does not meet the high demands of modern human life. Searching alternative anode materials with much higher specific capacity than graphite has thus attracted many researchers around the world in investigating new anodes of LIBs.

### 1.2. Silicon-Based Anode

Among many active material candidates for the anode of LIB, silicon exhibits a theoretically very high specific capacity of more than 4000 mAh/g, which is more than ten times of that of graphite-based anode. The followings barriers need to be overcome before silicon-based anode becomes a commercial product: (i) pulverization of silicon by up to 400% volume changes due to alloying and de-alloying of silicon with lithium; (ii) the formation of solid-electrolyte-interphase (SEI) on silicon; (iii) irreversible reactions of silicon with electrolyte to form compounds; (iv) the low conductivity of silicon; and (v) the loss of electrical connection between silicon and the current collector of the node.

Nanoscale silicon having high number ratios of surface atoms to bulk atoms is more capable of surviving the large volume changes. Many research groups have published a large number of papers related to anode made of silicon nanoparticles of sub-100 nm in size and nanostructured silicon-based anode. However, high costs are required to reduce the size of silicon particles. Small silicon particles have a large effective surface area to consume lithium for forming SEI [20]. Modification of silicon active materials including protective and conductivity enhancing coatings is of high priority towards developing an advanced anode for high-demanding future LIBs. Carbons, ultra-nano-crystalline-diamond (UNCD), silicon oxides, and silicon carbide are among investigated coatings on silicon.

### 1.3. Carbon Coatings

Graphitic carbon coatings on silicon provide electrical conductivity between silicon particles and with the current collector of the anode. Graphitic nanocarbons such as electrically conductive nanocarbon films, nanoparticles, fibers, CNTs, graphene, and graphitized polymers are examples [21,22,23,24,25,26,27,28,29,30,31,32,33,34,35,36]. We have reported plasma chemical vapor deposition (CVD) of sponge-like graphene-nanowall coatings on silicon particles, direct growth of graphitic nanocarbon films including CNTs on silicon flakes, and the application of thermally oxidized silicon for enhancing the physical integrity of silicon flakes. Anode performance is promising [15,16,37].

UNCD films consist of merged 2–5 nm diamond grains with grain boundaries filled with sp^2^ graphitic carbon-carbon bonds. UNCD exhibits chemical inertness like diamond, and possesses strong mechanical strength desirable for enhancing the physical integrity of UNCD-coated silicon-based anode. Lithium diffuses through diamond grains slowly but through graphitic grain boundaries with less resistance similar to the way it does in graphite. Because grain boundaries occupy only a small fraction of the surface area, the UNCD film spreads lithium ion current to avoid “hot spots” of ion current density. Silicon is less vulnerable to pulverization when its volume changes uniformly and less local stress is induced. Therefore, coatings with similar functions of UNCD are expected to enhance the physical integrity of silicon-based anode by means of its strong mechanical strength and by its current spreading capability. The chemical inertness of diamond is desirable, too. UNCD isolates silicon from reactions with the electrolyte to form lithium hexafluorosilicate (Li_2_SiF_6_) aggregates during cycling. The reaction is irreversible resulting in premature failure of LIBs [29,30]. Silicon oxide and silicon carbide in proper structures might play roles similar to UNCD.

### 1.4. Silicon Oxide for Silicon-Based Anode

Silicon oxide, SiO_x,_ where x is less than or equal to two, was investigated for improving stability of silicon-based anode [38,39,40,41,42]. The larger the x is, the harder the silicon oxide will be. On the other hand, the larger the x is, the smaller the overall charge storage capacity of the silicon oxide is. When high charge storage capacity and good physical integrity are compromised, SiOx survives a longer life than silicon of the same dimensions in the anode cycling. However, it exhibits lower ICE than pure silicon due to the formation of irreversible Li-salt. Furthermore, SiO_x_ exhibits higher electrical resistance than silicon. In order to take advantage of the cycling durability of SiO_x_, the value of x is optimized. A number of oxidized silicon materials were studied for anode applications. They include, for examples, (i) SiO-based anode materials; (ii) SiO_2_-based anode materials; (iii) non-stoichiometric SiO_x_–based anode materials; and (iv) Si-O-C based anode materials. Liu et al. published a comprehensive review of silicon oxides as a promising family of anode materials for lithium-ion batteries. References [38,39] reported relevant atomic models for amorphous Si, interfacial silicon sub-oxide, SiO_2_, and SiO.

SiO has the tendency of disproportionation into Si and SiO_2_ and restoring the high capacity of Si in a framework of SiO_2_. Although the overall capacity is lower than silicon, less volume changes make SiOx attractive for long-life LIB applications. Yang et al. studied the performance of SiO, SiO_0.8_, and SiO_1.1_ and found that the capacity decreases with increasing oxygen contents but with improving cycling performance and concluded that SiO_0.8_ had a reversible capacity of about 1600 mAh/g [40]. LIB anode containing SiOx, x < 2, is nowadays a commercial product.

SiO_2_ has a high theoretical specific capacity of 1965 mAh/g. However, SiO_2_ has high electrical resistivity and low diffusivity for Li+. Porous silicon oxide and nanoscale SiO_2_ partially overcome these problems. Jiao et al. synthesized 400 nm SiO_2_ balls by a sol-gel method. The initial specific capacity was 622 mAh/g with a low ICE being 54.8%. After discharge/charge for 500 cycles, the stoichiometry probably changed and some pure silicon contributes to an even higher specific capacity of 877 mAh/g. Liang et al. ball-milled SiO_2_ down to smaller than 1 μm in size and reported 600 mAh/g specific capacity after 150 cycles of discharges and charges [41,42].

The high manufacturing costs for SiOx makes the search for new materials and nanostructures of the same functions desirable. We recycled silicon flakes from by-products of silicon crystal and wafer manufacturing processes. The typical dimensions of an as-received silicon flake is about 100 nm in thickness and 800–1000 nm in width and length. Silicon flakes were oxidized to form SiO_2_ encapsulating the surface of individual silicon flakes. When these silicon flakes were broken into smaller pieces by high-energy ball milling, there are fresh silicon surfaces on the breaking planes. These exposed silicon surfaces provide windows for lithium to alloy with silicon [37].

### 1.5. Silicon Carbide for Silicon-Based Anode

Silicon carbide nanoparticles were reported by some research groups to be inactive to lithium but by other groups to be a promising active anode material [43]. Timmons et al. used high-energy ball milling to produce nanoparticles from Si-C composites and found the nanoparticles to be nanocrystalline SiC or silicon. The measured capacity was consistent with the contribution of 3750 mAh/g by alloying of silicon with lithium to form Si_15_Li_4_. These authors considered SiC to be an inactive anode material. Nevertheless, the presence of SiC did not block the diffusion of lithium for alloying with silicon [44]. On the other hand, thin-film phosphorus doped nanocrystalline 4H-SiC surrounded by amorphous SiC networks served as a stable active material of anode for LIB and exhibited a specific discharge capacity of 309 mAh/g for over 60 cycles at the C/10 (charge) rate and C/5 (discharge) rate. Kumari et al. showed that cubic (3C polytype) nano SiC, prepared by chemical vapor deposition (CVD), delivered a reversible lithium insertion capacity of about 1200 mAh/g over 200 cycles [45,46,47].

Sun et al. reported the use of twisted SiC nanofibers synthesized from resin-silica composites and containing 92.5 wt % cubic β-SiC and 7.5 wt % amorphous C as an anode active material. A stable reversible capacity of 254.5 mAh/g was retained for 250 cycles at a current density of 0.1 A/g. The same research group reported that after 500 cycles at 0.3 A/g, a high discharge capacity of 540.1 mAh/g was achieved. This is higher than the theoretical capacity of graphite [48].

Intrinsic crystalline SiC is highly resistive at room temperature. Nanoscale SiC, such as SiC nanofibers and amorphous silicon carbide, are capable candidates as anode active materials although the capacity is lower than that of graphite and silicon. Proper coatings on crystalline SiC are necessary to enhance the electrical conductivity.

Besides charge storage capacity, silicon carbide may exhibit other favorable functions for silicon-based anode. In 2019, Yu et al. proposed and demonstrated the application of SiC as encapsulation around silicon nanoparticles to isolate silicon from generating lithium hexafluorosilicate (Li_2_SiF_6_) aggregates by reactions with lithium hexafluorophosphate (LiPF_6_) electrolyte during cycling. Some carbon coatings directly deposited on silicon for enhancing conductivity do not protect silicon but, instead, serve as catalysts for accelerating the undesirable silicon reactions with the electrolyte. Yu et al. took advantage of the high strength and toughness of silicon carbide (SiC) coating between silicon and a conductivity enhancing carbon coating and retained a specific capacity of 980 mAh/g at a current density of 1 A/g after 800 cycles with ICE being higher than 88.5%. The Si@SiC@C nanoparticles were 100 nm in size. The thicknesses of the carbon and SiC layers were approximately 8–10 and 7–8 nm, respectively. The raw silicon particles were about 80 nm in size. Carbon coating was heat-treated at 850 °C in a gaseous mixture of Ar and C_2_H_4_ for 35 min. Si@C was calcined at 1300 °C for 1 h in a gas mixture of Ar and H_2_. The carbon coating process repeats to coat a conductive carbon layer on top of the Si@SiC particles. The performance is promising. The processing involves high temperature up to 1300 °C while the raw material consists of 80-nm silicon nanoparticles [49].

From the viewpoint of mass producing silicon active materials for LIB anode applications, low-temperature processing using inexpensive raw silicon is attractive. In our work, the processing temperature is limited to 1000 °C and recycled silicon flakes from wastes of silicon wafer manufacturing processes were used as the raw silicon material.

## 2. Materials and Methods

### 2.1. Chemicals

AUO Crystal Corporation in Taichung City, Taiwan, ROC supplied silicon flakes of about 100 nm thick and 1000 nm in length and width. It is a silicon wafer manufacturing company in Taiwan. The silicon flakes are part of silicon containing waste slurry generated by cutting silicon ingots and from other silicon wafer manufacturing processes. The company uses an economic and proprietary chemical process to recover and purify the silicon flakes from the slurry. Battery-grade electrolyte, i.e., 1M LiPF6 dissolved in ethylene carbonate (EC) and diethyl carbonate (DEC) in a 1:1 ratio by volume was purchased from Yichun Jinhui New Energy Materials Co. (Taipei, Taiwan). Resorcinol, cetyltrimethylammonium bromide (CTAB, 98%), and formaldehyde (37% solution) were purchased from Sigma-Aldrich (Taipei, Taiwan). We used as-received chemicals without further purification.

### 2.2. Synthesis of SiC

Silicon carbide was synthesized using a low-pressure chemical vapor deposition (CVD) reactor made of a two-inch-diameter quartz tubing. After an initial purge by argon (500 sccm) and hydrogen (10 sccm) at 800 °C, the reactor temperature increased to 1000 °C. A mixture of argon (500 sccm), hydrogen (10 sccm), and methane (20 sccm) was fed into the reactor. The gas pressure was set at 50 torr when the reactor temperature increased from 800 to 1000 °C. The reaction time was 1 h or 5 h. The reactor cooled down in argon environments.

### 2.3. Deposition of RF Carbon Coatings

Silicon flakes (8 g) was sequentially mixed with ammonium hydroxide aqueous solution (6.67 mL), D.I. water (900 mL), and an aqueous solution of CTAB (66.67 mL, 0.01 M), and vigorously stirred for 30 min to ensure the complete adsorption of CTAB on the silicon flake surface. We mixed the product with 3.33 g of resorcinol and 4.7 mL formaldehyde solution. The mixture was stirred for 16 h and RF-coated silicon flakes were collected. We heated the RF coating at 800 °C for 4 h under argon environments to form conductive RF carbon coatings [50,51].

### 2.4. Materials Characterization

The morphology and structure of the nanocarbon-coated silicon were observed by means of scanning electron microscopy (SEM, Hitachi-SU8000, Taipei, Taiwan). Scanning Transmission Electron Microscopy (JEOL JEM-2100F Cs STEM, Taipei, Taiwan) was used to reveal the silicon carbide layer on a silicon flake. The acceleration voltage was 200 kV. A Horiba Scientific (Taipei, Taiwan) Raman system with a green laser at 532 nm and laser power at 450 mW was used to measure Raman spectra. The laser beam was focused on the sample surface in an area of about 10 µm in size. Raman spectra reveals the nanostructures of the samples. XRD (Bruker AXS Gmbh, Karlsruhe, Germany) was used to analyze the crystalline structure of the Si@SiC flakes. FTIR measurements were conducted on a Thermo/Nicloet, FTIR transmission spectrometer (Thermo Fisher Scientific, Taipei, Taiwan) at room temperature under N_2_ flow with a resolution of 4 cm^−1^, spectral range 400–4000 cm^−1^, using the KBr method.

### 2.5. Resistance Measurements

Two probes of a resistance meter are connected to two copper electrodes of 5 mm wide at a distance of 1 cm pressing onto two sides of a pile of silicon powder between two glass plates. This measurement is for a quick evaluation of the conductivity of carbon coatings. The order of magnitude in resistance guides us in adjusting the fabrication parameters for optimization. Accurate electrochemical impedance spectroscopy is applied to measure the impedance after anode is made.

### 2.6. Test Cells

Coin half-cells (CR2032) were fabricated for testing of the performance of the anode. Nanocarbon-coated silicon flakes were mixed with carbon black, sodium carboxymethyl cellulose (NaCMC), and Styrene-Butadiene Rubber (SBR) with a weight ratio of 6:2:1:1. The slurry was stirred homogeneously before being coated to a 10-μm thick copper foil by a doctor blade. The thickness of the active materials was 15 µm. After the electrode dried at 60 °C for 12 h, the electrode was cut into small pieces with a diameter of 12 mm. The electrode was placed in an Argon-filled glove box with residual oxygen and moisture contents of less than 0.5 ppm to assemble coin cells. The electrolyte was the 1 M LiPF6 in ethylene carbonate (EC) and dimethyl carbonate (DMC) (1:1 *v*/*v*) solution.

### 2.7. Characterization of Test Cells

The charge/discharge cycling performance was analyzed by a battery testing system (BAT-750B). The specific capacity refers to the mAh per gram of silicon active material in the anode. The discharging cuts off at the potential of 10 mV and the charging cuts off at the potential of 1.5 V. Cyclic voltammetry (CV) was measured using Autolab (Metrohm AUTOLAB BV). The CV measurements used a scanning rate of 0.1 mV/s at the room temperature.

## 3. Results and Discussion

**Figure 1:**
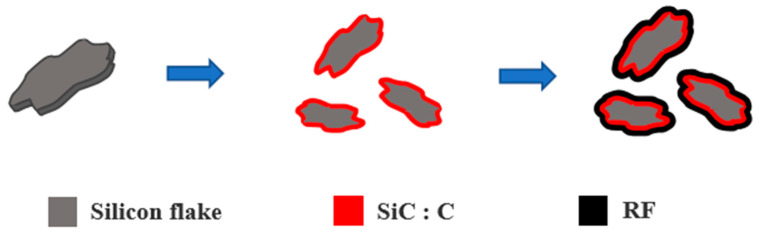
Schematic diagrams of an example of forming a layer of silicon carbide on the surface of a silicon flake followed by the coating of a layer of Resorcinol–Formaldehyde (RF) electrically conductive carbon coating.

Figure 1 shows schematically that silicon carbide (SiC) with a nanocarbon over-layer was formed on the surface of a thin and flat silicon flake of about 100 nm thick and 1000 nm long and wide. The silicon flakes are subsequently coated with Resorcinol–Formaldehyde (RF) carbon layers. SiC protects silicon from excessive reactions with the electrolyte to form irreversible solid-electrolyte-interphase (SEI) layers and other compounds on silicon surface. A thin layer of SiC and nanocarbons deposits on Si surface after a 5-h thermal treatment in a gas mixture of methane and hydrogen at 1000 °C. In order to increase the electrical conductivity of the silicon active material in the anode, an additional layer of RF carbon was deposited on the surface layer of a SiC-coated silicon flake. RF carbon is chosen because of the simple and economic solution-based coating process. Anodes made of pristine silicon flakes and those with RF carbon coatings, SiC coatings, and combined SiC and RF carbon coatings were studied.

**Figure 2:**
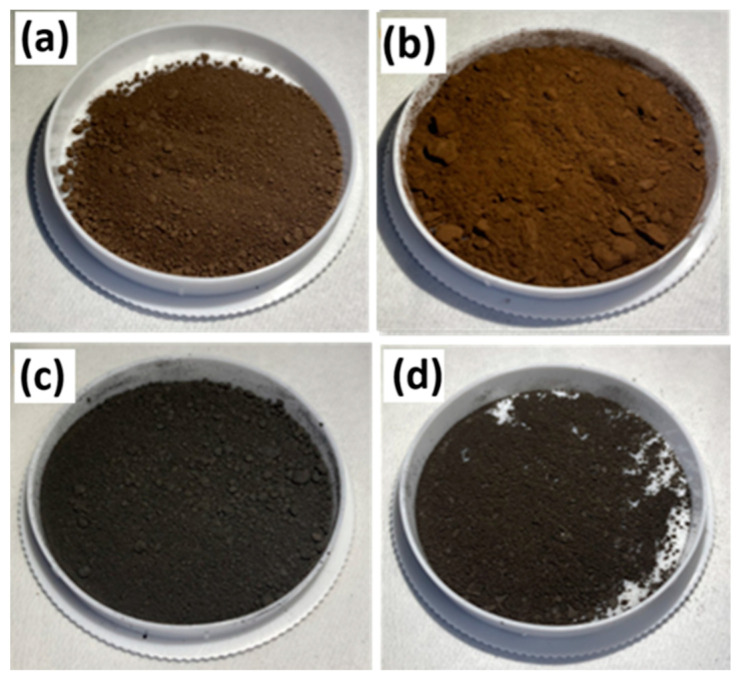
Photographs of (**a**) pristine silicon flakes and silicon flakes with (**b**) RF carbon coatings; (**c**) SiC; and (**d**) combined SiC and RF carbon coatings.

Figure 2a shows photographs of dark brownish pristine silicon flakes. Figure 2b shows silicon flakes with a coating of RF carbon. The color became light brown. Figure 2c shows that five-hour reactions converted the silicon flakes into black flakes with SiC and nanocarbon coatings. Figure 2d shows that an RF carbon over-layer on SiC and nanocarbon-coated silicon flakes exhibits between gray and black color indicating the color of the RF carbon over-layer.

**Figure 3:**
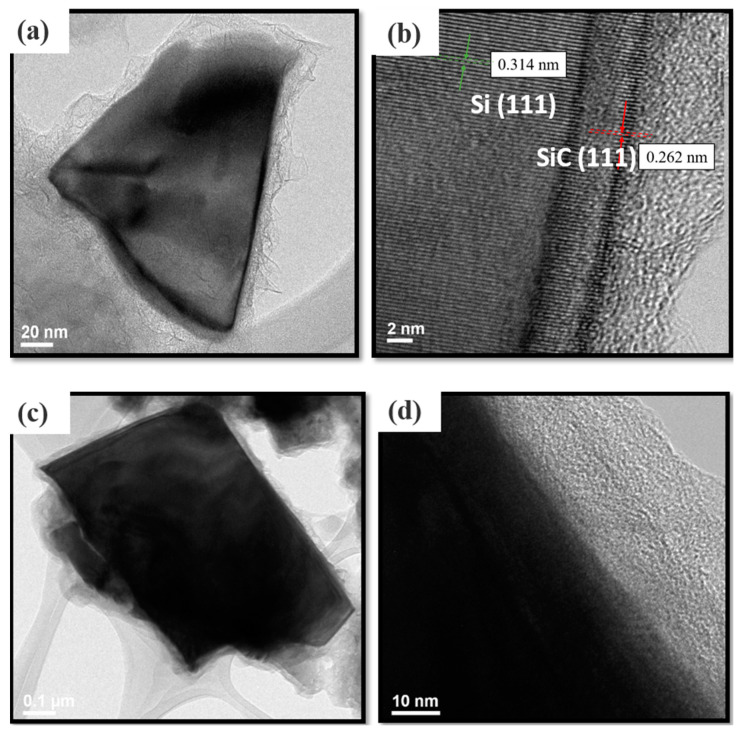
TEM images of a SiC coating on a pristine silicon flake (**a**,**b**) and an RF coating on SiC-coated silicon flake (**c**,**d**). Thermal carbonization of silicon flakes for SiC synthesis lasted for 5 h.

Figure 3a shows a high-resolution TEM image of a silicon flake, which reacted with the gas mixture of methane and hydrogen at 1000 °C for 5 h. The TEM image displays an overlayer of nanocarbons. Figure 3b shows that a crystalline SiC (111) of about 5 nm thick was grown on crystalline Si (111). On the SiC a nanocarbon layer of about 10 nm thick is mixed with amorphours carbons. Silicon flakes with SiC and nanocarbons coatings were measured to exhibit a electrical resistance of a few hudreds to a few thousand Ohms. For simple description in the following discussion, “SiC coating” will refer to a combined coating of a 5-nm SiC layer and a 10-nm nanocarbon layer formed on a silicon flake by the 5-h thermal carbonization process.

Figure 3c shows a high resolution TEM image of a SiC-coated silicon flake with an RF carbon layer with a thickness of about 30 nm. Figure 3d shows that the RF carbon layer is amorphous. The RF carbon coatings on silicon flakes with SiC coatings were measured to exhibit a lower electrical resistance of tens of Ohms comapared to hundreds to thousands of Ohms measured for silicon flakes with only SiC coatings. It shows that the the SiC-coated silicon flakes active material gains improved electrical conductivity by the additional RF carbon coatings. In the following discussion, the RF carbon coatings on SiC-coated silicon flakes will be referred to as silicon flakes with combined SiC and RF carbon coatings.

**Figure 4:**
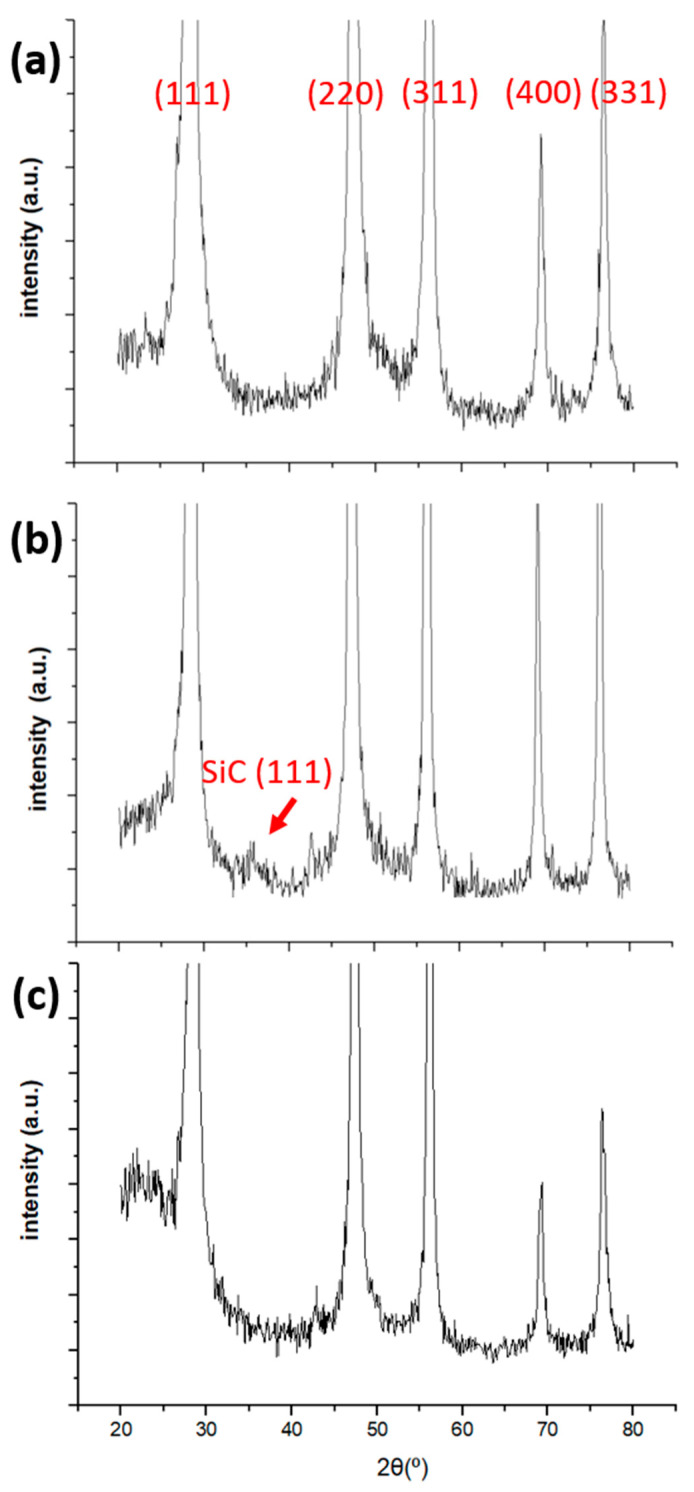
X-ray diffraction patterns of (**a**) pristine silicon flakes, (**b**) silicon flakes after 5-h thermal carbonization, and (**c**) silicon flakes after 1-h thermal carbonization.

We applied an X-ray diffractometer to analyze the SiC layer formed on silicon flakes after 5-h thermal carbonization of silicon. A small SiC (111) peak is displayed in Figure 4b compared with that in Figure 4a for pristine silicon flakes. The SiC layer is about 5 nm thick. Due to the sensitivity limit of XRD analysis for a thin layer of SiC on silicon flakes, the SiC peak is weak but distinguishable. When the thermal carbonization process is shortened to 1 h, there is no SiC (111) peak like the one shown in Figure 4b. The SiC(111) XRD peak for SiC-coated silicon flakes, when combined with the TEM image shown in Figure 3b, confirms that crystalline SiC forms on silicon flakes after 5-h thermal carbonization of silicon. SiC coatings will refer to 5-h SiC coatings on pristine silicon flakes in the following discussion.

**Figure 5:**
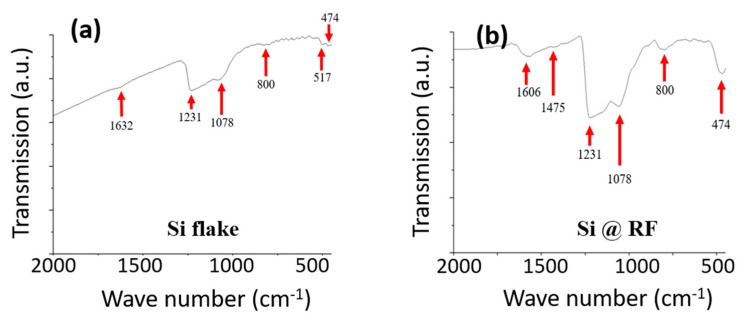
FTIR spectra of (**a**) pristine silicon flakes and (**b**) pristine silicon flakes with RF carbon coatings.

Figure 5 shows FTIR spectra of silicon flakes and silicon flakes with RF carbon coatings. Comparing Figure 5a with Figure 5b, the main difference is an absorption band at 1606 cm^−1^. This absorption band corresponds to carbon-carbon double bonds. It shows that the RF carbon coating contains graphitic carbon-carbon bonds although the TEM image shows that it is an amorphous coating [52,53]. High electrical conductivity of the RF carbon coating is attributed to graphitic carbons.

**Figure 6:**
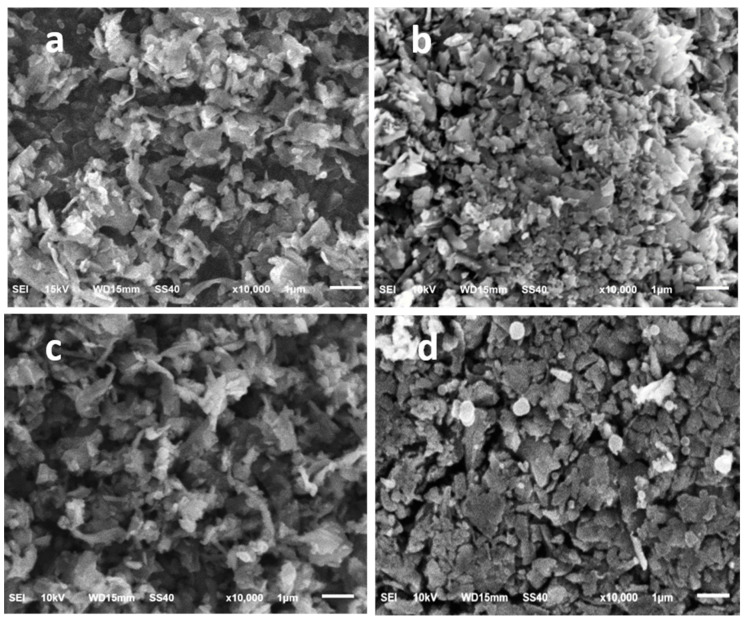
SEM images of (**a**) silicon flakes, (**b**) silicon flakes with an RF carbon coating, (**c**) silicon flakes with a layer of SiC and nanocarbon (5 h-process), and (**d**) silicon flakes with a layer of SiC and nanocarbon (5-h process) followed by the coating of a layer of RF carbon.

Figure 6 shows SEM images of (a) pristine silicon flakes; (b) silicon flakes with RF carbon coating; (c) silicon flakes with SiC coating; (d) silicon flakes with combined SiC and RF carbon coatings. Pristine and SiC-coated silicon flakes look similar in their SEM images with plenty of pores. RF carbon coatings add more thickness and lateral dimensions to the pristine silicon flakes. There are smaller pores between silicon flakes with RF carbon coatings than those without RF carbon coatings.

**Figure 7:**
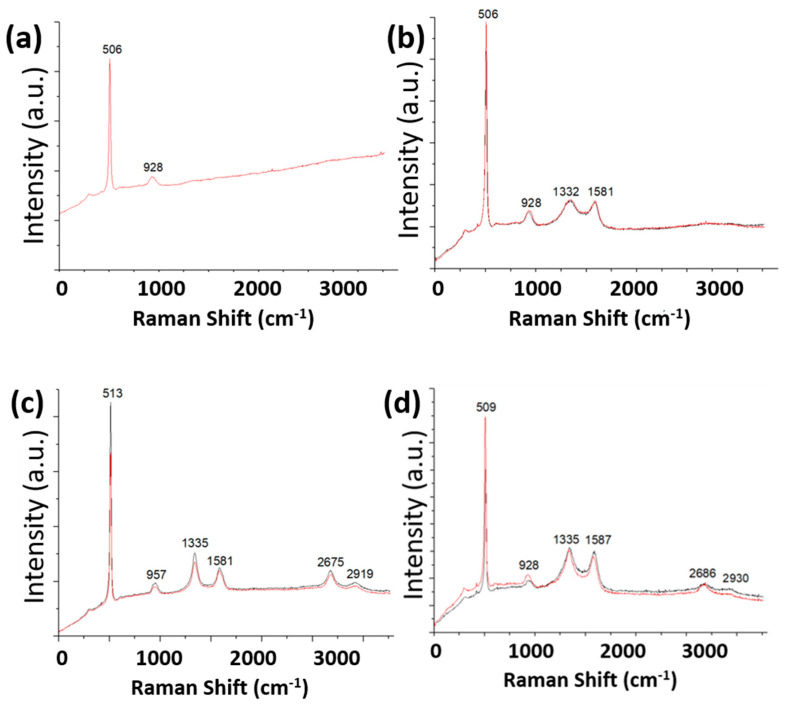
Raman spectra excited by 532 nm laser measured from (**a**) silicon flakes, (**b**) silicon flakes with an RF carbon coating, (**c**) silicon flakes with SiC coatings, and (**d**) silicon flakes with combined SiC and RF carbon coatings.

Figure 7 shows Raman spectra for pristine silicon flakes and silicon flakes with SiC coatings, RF carbon coatings, and their combinations. A 532-nm laser excites Raman scattering of the silicon flakes with coatings. By comparing the spectrum in Figure 7b for RF-carbon-coated silicon flakes with that in Figure 7a for pristine silicon flakes, the RF-carbon coatings exhibit a broader D-band and a sharper G-band besides the silicon Raman peaks. Both spectra do not exhibit a 2D-band. G-band Raman signal around 1580 cm^−1^ is from sp^2^ graphitic carbon. Crystalline sp^2^-bonded carbon also exhibits a 2D-band Raman scattering signal around 2670s cm^−1^ from double resonance with two phonon interactions. D-band in the range of 1330s–1350s cm^−1^ is from defective crystalline carbons. The RF carbon coatings consist of sp^2^ bonded carbons but are highly defective. This is consistent with the TEM image of an amorphous RF carbon film, the measured FTIR absorption by carbon-carbon double bonds, and the low electrical resistance of silicon flakes with RF carbon coatings. RF carbon coating provides electrical conductivity for silicon flakes.

Figure 7c shows a Raman spectrum for silicon flakes with SiC coatings. Both the Raman G-band and the D-band for silicon flakes with SiC coatings shown in Figure 7c are narrower than those for RF carbon coatings shown in Figure 7b. The signal intensity ratio of the D-band to the G-band is inversely proportional to the crystalline quality. The D-band signal strength is stronger than that of the G-band. The Raman scattering signal in the wavenumber range between the D-band and the G-band is weak. The Raman spectrum also exhibits clear 2D-band Raman signal. These Raman features are characteristic of a defective crystalline carbon film that forms by thermal carbonization at 1000 °C in methane and hydrogen environments.

The Raman spectrum shown in Figure 7c also exhibits a Raman peak at 957 cm^−1^. This is attributed to a combined Raman signal from the longitudinal optical (LO) mode of vibration in SiC and a shifted double resonance vibration of silicon from 928 cm^−1^ due to SiC-Si interfacial stress. The Raman shift of the silicon peak from 506 cm^−1^ shown in Figure 7a to 513 cm^−1^ is shown in Figure 7c. The Raman shift is mainly attributed to the interfacial stress due to lattice mismatching heteroepitaxial growth of SiC on Si [54,55,56,57,58].

Figure 7d shows Raman spectra of silicon flakes with combined SiC and RF carbon coatings. After the RF carbon coating and the subsequent thermal treatment of the 30 nm thick RF coatings, the shift of silicon Raman peaks shown in Figure 7c is restored. The interfacial stress might have relaxed during the thermal treatment of RF carbon coatings. The Raman signal strength in the wavenumber range between the D-band and the G-band increases from that with only SiC coatings. The 2-D band is weaker than that for only SiC coatings but displays clearly. The nanocarbon layer grown on top of SiC during the thermal carbonization process is attributed to this 2-D band Raman scattering signal.

**Figure 8:**
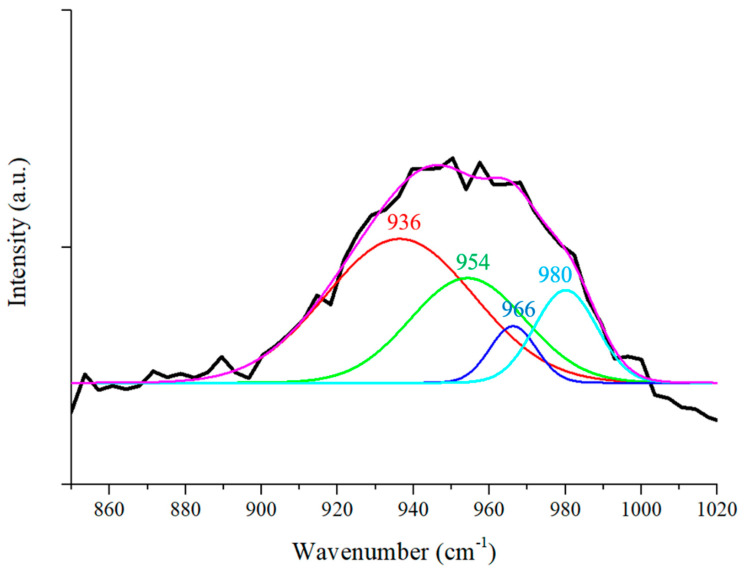
Gaussian curve fitting of the Raman Peak at 957 cm^−1^ in Figure 7c. Peaks 936, 954, and 988 cm^−1^ correspond to Si. The Raman band at 966 cm^−1^ is attributed to Raman scattering of SiC.

Figure 8 shows four Gaussian curves fitting the 957 cm^−1^ Raman peak measured from silicon flakes coated with SiC for 5 h. Peaks 936, 954, and 980 cm^−1^ correspond to Si. Raman shift at 966 cm^−1^ corresponds to Raman scattering from SiC crystals. The SiC peak is complementary to the TEM image shown in Figure 4b to show the presence of crystalline SiC grown on the surface of silicon flakes [59,60].

**Figure 9:**
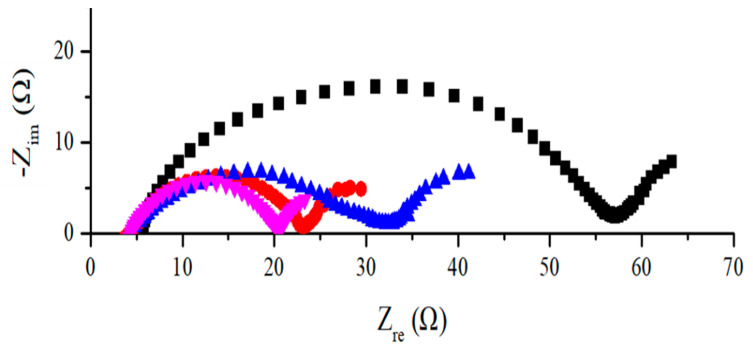
Electrochemical impedance spectra of anode before discharge/charge cycling experiments measured from four sets of anodes made of (black square) silicon flakes, (red dots) silicon flakes coated with an RF carbon layer, (blue triangle) silicon flakes coated with SiC, and (pink reverse triangle) silicon flakes with combined SiC and RF carbon coatings.

Figure 9 shows electrochemical impedance spectra of silicon-flake-based anodes. The measured impedance increases in the order of silicon-flake-based-anodes with (i) combined SiC and RF carbon coatings; (ii) RF carbon coatings; (iii) SiC coatings; and (iv) pristine silicon flakes. RF carbon coatings provide excellent improvement of the impedance of pristine silicon flakes. SiC improves the impedance of pristine silicon flakes but not as much as RF carbon coatings. The combined SiC and RF carbon coatings exhibit the lowest electrochemical impedance. SiC coatings and RF carbon coatings form a parallel-connected conductor leading to the lowest impedance.

**Figure 10:**
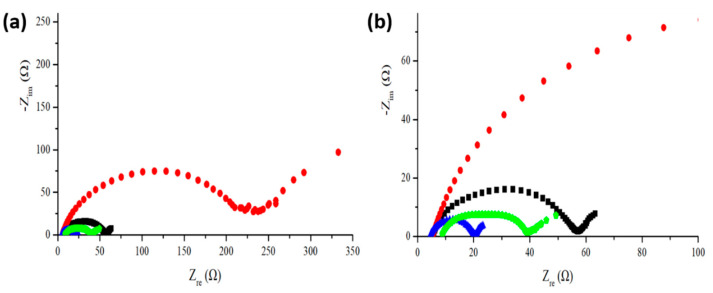
(**a**) Electrochemical impedance spectra measured from as-fabricated anode and after 150 cycles of discharge/charge at 0.2 C for anode made of (black square) pristine silicon flakes, (red dots) pristine silicon flakes after cycling, (blue triangle) silicon flakes with a combined SiC and RF carbon coatings, and (green pentagon) silicon flakes with a combined SiC and RF carbon coatings after cycling. (**b**) Enlarged spectra shown in (**a**).

Figure 10 shows the electrochemical impedance spectra (EIS) of anodes made of pristine silicon flakes and silicon flakes with combined SiC and RF carbon coatings. We measured EIS for as-fabricated anodes and those after 150 times of discharge/charge cycling at a rate of 0.2 C. The pristine-silicon-flake-based anode failed before the end of the 150 discharge/charge cycles. Figure 10b shows low impedance retained by silicon flakes with combined SiC and RF carbon coatings at the 150th cycle of discharge/charge. The impedance increases by about two times after 150 cycles of discharge/charge operation. At the end of 150 cycles, anode with combined SiC and RF carbon coatings exhibits impedance even lower than that made of pristine silicon flakes before cycling.

**Figure 11:**
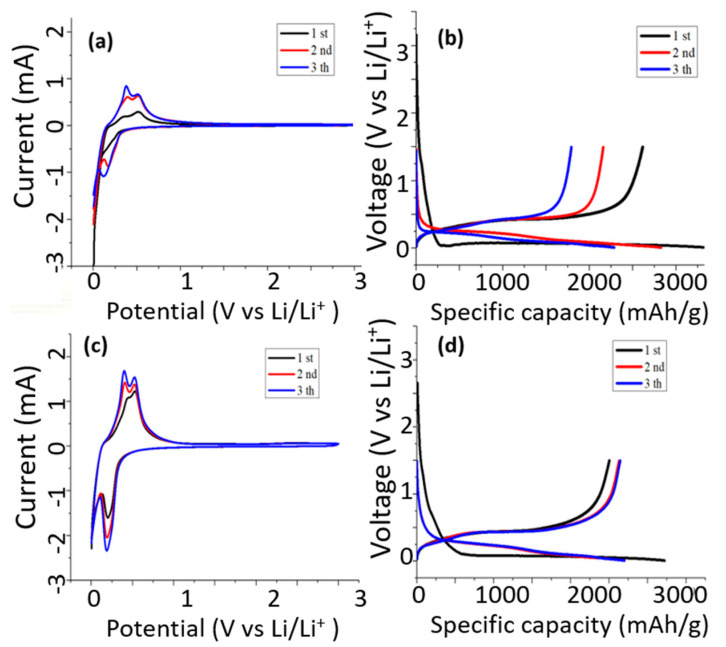
Cyclic voltammetry measurements of anodes made of (**a**,**b**) pristine silicon flakes and (**c**,**d**) silicon flakes with combined SiC and RF carbon coatings. The scan rate for (**a**,**c**) is 0.1mV/s. The discharge/charge rate for (**b**,**d**) is 200 mA/g.

Figure 11 shows cyclic voltammetry (CV) measurement results of anodes made of Figure 11a,b pristine silicon flakes and Figure 11c,d silicon flakes with combined SiC and RF carbon coatings. The scan rate for measurements shown in Figure 11a,c is 0.1mV/s. The discharge/charge rate is 200 mA/g for the measurements shown in Figure 11b,d. Figure 11a,c shows similar discharge/charge CV curves except that the pristine silicon-flake-based anode exhibits much lower currents due to the high resistance of silicon flakes without carbon coatings. With combined SiC and RF carbon coatings, silicon flakes are more conductive. The currents increase significantly. For both cases, the discharge begins with the lithiation of the crystalline silicon at near zero volt. The lithiation forms lithium-silicon alloys. When alloys dissociate to release lithium, silicon does not recrystallize but instead becomes amorphous silicon. The delithiation curves exhibit two peaks near 0.4 V and 0.6 V. These peaks are attributed to a two-step delithiation process, i.e., the anode first delithiates to become an intermediate lithium-silicon alloy. A full dissociation of the alloy makes it become amorphous silicon. Figure 11b shows that the anode made of pristine silicon flakes discharges to a level exceeding 3000 mAh/g during the first half cycle. However, both the discharge and charge levels decay rapidly during the first three cycles. Figure 11d shows that the anode made of silicon flakes with combined SiC and RF carbon coatings exhibits a more constant initial capacity of the charging curves than that of anode made of pristine silicon flakes. The ICE of the anode made of pristine silicon flakes is 74.5% while that of the anode made of silicon flakes with combined SiC and RF carbon coatings is also 74.5%. The RF carbon coatings on the outer surface of silicon flakes improve conductivity but does not improve the ICE. Optimization of the conductivity enhancing RF carbon coatings or using different conductivity enhancers with low reactivity with the electrolyte to form irreversible lithium containing compounds is desirable.

**Figure 12:**
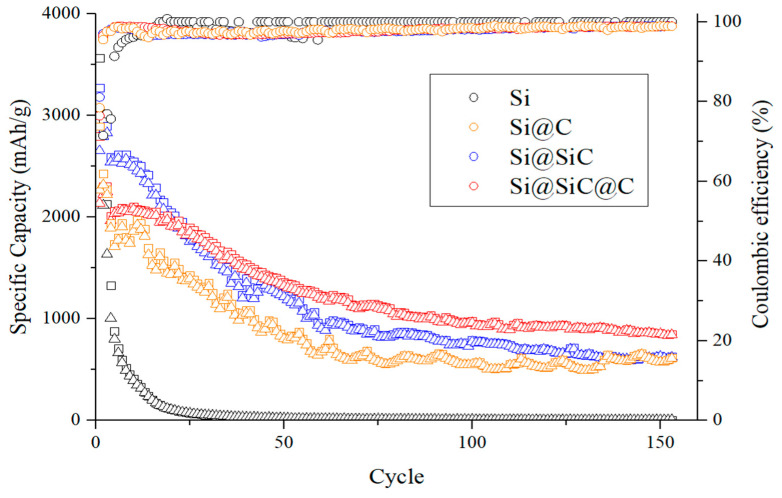
Cycling performance of anodes made of (black) pristine silicon flakes, (orange) silicon flakes with an RF carbon coating, (blue) silicon flakes with SiC coatings, (red) silicon flakes with combined SiC and RF carbon coatings.

Figure 12 compares discharge/charge cycling performance of anodes made of (black) pristine silicon flakes, (orange) silicon flakes with RF carbon coatings, (blue) silicon flakes with SiC coatings, and (red) silicon flakes with combined SiC and RF carbon coatings. The discharge/charge rate for the first three cycles is 0.05 C and the remaining cycles are 0.12 C. The ICE is 74.5%, 79.7%, 80.36%, and 74.5% corresponding to the black, orange, blue, and red charts, respectively. Anodes with coatings on silicon flakes exhibit much improved capacity retention after 150 cycles. The rate of decay of capacity of the red and orange charts slows down after 100 cycles while the blue chart starts at a high capacity but continues to decay more quickly. Both the red chart and the orange chart correspond to anode made of silicon flakes with the outmost layers being RF carbon coatings. The conductivity of RF carbon coatings is important to keep silicon connected to the current collector of the anode so that silicon can retain its charge and discharge capacity. The blue and red charts exhibit the slower decay in capacity in the first 100 cycles. This is attributed to improved physical integrity provided by SiC coatings and reduced reactions by silicon with the electrolyte by the SiC encapsulation of silicon flakes.

The slightly increased diffusion resistance by SiC coatings for lithium to alloy with silicon makes the SiC coatings act as an effective lithium diffusion spreader. Lithium reacts with silicon from all directions around a silicon flake more uniformly when there is a SiC diffusion spreader. The reaction between lithium and silicon forms alloys with significant volume expansion. If alloying occurs in one area of a silicon flake faster than others do, that area will expand faster than the rest of the silicon flake. This creates internal stress that may accelerate the pulverization of a silicon flake. Therefore, SiC plays a role to prevent local high-density lithium ion flux. The spreader minimizes local hot spots where lithium diffuses into and reacts with silicon much faster than other areas. When alloying reactions proceed uniformly from all directions of a silicon flake, the alloyed silicon flake expands in volume uniformly in a more balanced mode. Less internal stress forms and the physical integrity is preserved.

By encapsulating a silicon flake with SiC also helps reducing the chances of silicon from reactions with the electrolyte to form lithium containing irreversible compounds. This is especially important for preserving the fixed amount of lithium contents in a packaged full battery. These effects play out well according to the cycling charts shown in Figure 12. At the end of 150 cycles, the red cycling chart shows that the silicon flakes with combined SiC and RF carbon coatings retained the most capacity. The capacity stays above 850 mAh/g for 150 cycles. We do not know the exact mechanisms responsible for the decay of the capacity from 2000 mAh/g to 850 mAh/g yet. Imperfection of interfaces between the silicon flakes, SiC coatings, and RF carbon coatings might be the cause. The amorphous nanocarbons formed during the 5-h thermal carbonization of silicon might have adverse effects on the interfacial properties between SiC and RF carbon coatings. We are carrying out further research along this direction and will be reporting new results in the near future.

**Figure 13:**
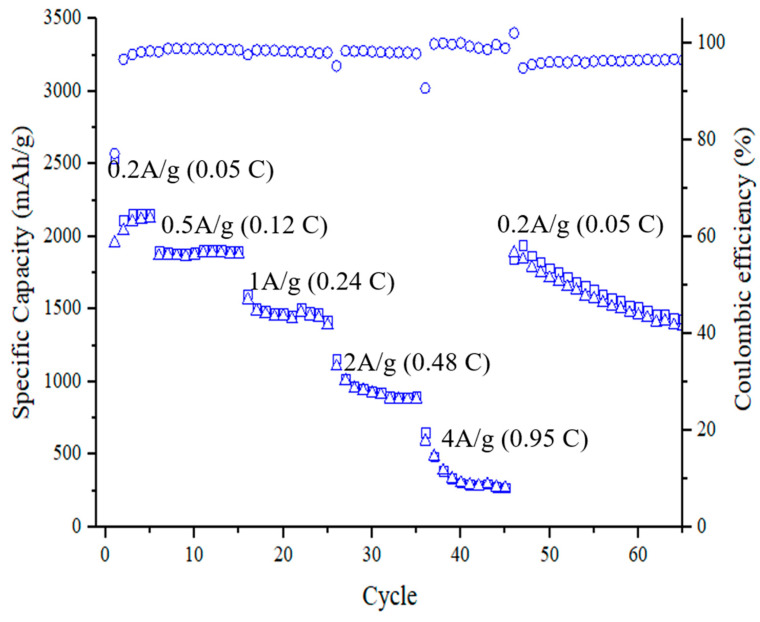
Cycling performance of the specific capacity and Coulombic efficiency of an anode made of silicon flakes with combined SiC and RF carbon coatings at discharge/charge rates ranging from 0.2 A/g (0.05 C) to 4 A/g (0.95 C). The discharge/charge rate is based on the total weight of active materials consisting of silicon flakes and combined SiC and RF carbon coatings.

Figure 13 shows cycling performance of the specific capacity and Coulombic efficiency of an anode made of silicon flakes with combined SiC and RF carbon coatings at discharge/charge rates ranging from 0.2 A/g (0.05 C) to 4 A/g (0.95 C). Rates (per gram) are calculated based on the total weight of silicon flakes and surface coatings. The rate for the first three cycles is at 0.01C. For each rate, ten cycles are performed. At 0.5 A/g, the capacity is between 1750 and 2000 mAh/g. For a higher discharge/charge rate at 1 A/g, the capacity remains between 1250 and 1500 mAh/g. At the rate of 2 A/g, the capacity remains between 750 and 1000 mAh/g. When the rate goes back to 0.2 A/g, the capacity restores to a level between 1750 and 2000 mAh/g. These cycling results show that silicon flakes with combined SiC and RF carbon coatings are effective in enabling a long cycling life and high-capacity silicon-flake-based anode of LIB.

Table 1 summarizes the initial Coulombic efficiency (ICE) and specific capacity measured at 100th and 150th cycles of discharge/charge operation of anodes made of (i) pristine silicon flakes; (ii) silicon flakes with RF carbon coatings; (iii) silicon flakes with SiC coatings; (iv) silicon flakes with combined SiC and RF carbon coatings, and (v) pre-lithiated anode of (iv). The discharge/charge rate is 0.2 A/g for the initial three cycles and 0.5 A/g for the remaining cycles. Pristine silicon flakes and those with RF carbon coatings alone exhibit poor capacity retention. SiC-coated anode exhibits the highest ICE but only a modest capacity retention. We attribute the high ICE to reduced reactions between silicon and the electrolyte due to encapsulation of silicon flakes by SiC coatings. It reduces reactions between silicon and electrolyte in forming irreversible lithium-containing compounds. Anode made of silicon flakes with SiC coatings but without RF carbon coatings exhibits low capacity retention. This is attributed to the low electrical conductivity of SiC-coated silicon flakes. For the anode made of silicon flakes with combined SiC and RF carbon coatings, the capacity retention increases to 955 mAh/g at the 100th cycle and 843 mAh/g at the 150th cycle. However, the ICE deceases from 82.2% to 76.4% due to the additional RF carbon coatings. The conductivity enhancing carbon coatings need further optimization to reduce reactions with the electrolyte in forming irreversible compounds.

We pre-lithiated anodes made of silicon flakes with combined SiC and RF carbon coatings by means of the process reported in [61]. A lithium foil served as the cathode with a separator placed between the cathode and the anode that is to be pre-lithiated. Electrolyte fills the space between the separator and both the cathode and the anode. A resistor is connected between the cathode and the anode. Electron current flows from lithium foil through the resistor to the current collector of the anode and then to silicon flakes in the anode. Lithium ions diffuses from the cathode through the electrolyte and the separator to reach the anode for combining with electrons and complete the pre-lithiation process. We did this in an argon-filled glove box. Voltage drop across the resistor between the anode and the cathode is measured for determining when to terminate the pre-lithiation process. As shown in Table 1, the ICE increased from 76.4% to 97.1%. The capacity retention remains about the same as that without pre-lithiation and exhibits 970 mAh/g at 100th cycle and 774 mAh/g at 150th cycle.

Cycling performance of this work is compared with three references [49,62,63]. The combined SiC and RF carbon coatings for silicon-flake-based anode exhibited comparable cycling performance with reports of references [62,63]. Silicon nanoparticles of 50–120-nm were used for the referenced results. Voids were formed between the conductivity enhancing carbon coatings and the silicon cores. Our work uses much larger silicon flakes of 100 nm thick and 1000 nm in lateral dimensions without additional void-forming processes. All referenced cases used FEC additive in the electrolyte. FEC is relatively expensive but improves the cycling performance of silicon-based anode [64,65]. Our electrolyte for this work did not include FEC. The ICE reported in [49] was superior to data presented in the work with the capacity retention being slightly better. The work of reference [49] used 80-nm silicon nanoparticles, FEC additive to the electrolyte, and thermal carbonization of silicon at 1300 °C for SiC. Our work applied 1000 °C for carbonization of silicon to form SiC coatings. Reference [49] added 5% by weight of CNTs as a conductivity-enhancing agent. Our work used Super-P carbon black as a conductivity-enhancing agent. Highly graphitic CNTs are less active than carbon black in forming irreversible compounds by reactions with the electrolyte and thus are more favorable for achieving a high ICE. More research on the addition of FEC in the electrolyte and further optimization of the conductivity enhancing additives and nanocarbon coatings on silicon flakes as an active anode material is being undertaken and will be reported elsewhere in the near future.

## 4. Conclusions

We coated silicon flakes of about 100 × 1000 × 1000 nm in size, which were recycled from wastes of silicon wafer manufacturing processes with combined silicon carbide (SiC) and graphitic (Resorcinol–Formaldehyde (RF)) carbon coatings. Silicon flakes served as the active materials of LIB anode. Thermal carbonization of silicon at 1000 °C for 5 h forms 5-nm SiC encapsulating silicon flakes. SiC provides physical strength to help with maintaining integrity of silicon flakes and isolate silicon from irreversible reactions with electrolyte. RF carbon coatings provide enhanced electrical conductivity for SiC-encapsulated silicon flakes. Silicon-flake-based anode with combined SiC and RF carbon coatings exhibits an ICE of 76% and retains a specific capacity of 955 mAh/g at 100th cycle and 850 mAh/g at 150th cycle of repetitive discharge and charge operation at 0.2 C. Pre-lithiation of the anode increases the ICE from 76% to 97%. The medium high diffusion resistance of SiC for lithium in comparison with that of silicon makes the SiC encapsulation serve as a diffusion spreader for more uniform lithium-silicon alloying reactions in all directions from the surface of a silicon flake. As a result, the probability of pulverization of a silicon flake decreases and the durability of silicon-flake-based anode improves significantly. Further optimization of the anode may lead to an economic recycling process for mass production of silicon-flake-based anode of LIBs.

## Figures and Tables

**Table 1:** Performance summary of silicon-flake-based-anodes.

Anode Active	Current	Mass Loading	ICE	100th Cycle	150th Cycle
Material	(A/g)	(mg/cm2)	(%)	Capacity	Capacity
				(mAh/g)	(mAh/g)
Si	0.2–0.5	2.03	78.3	9	6
Si@RF	0.2–0.5	1.68	78.9	665	474
Si@SiC	0.2–0.5	2.04	82.2	766	623
Si@SiC@RF	0.2–0.5	1.50	76.4	955	843
Si@SiC@RF + Pre-Li	0.2–0.5	1.50	97.1	970	774
